# Supplementary figures and images for: Quantitative Proteomics Reveals Molecular Network Driving Stromal Cell Differentiation: Implications for Corneal Wound Healing
Source: Int J Mol Sci. 2022 Feb 25;23(5):2572. doi: 10.3390/ijms23052572 (PMC8910342; doi:10.3390/ijms23052572)

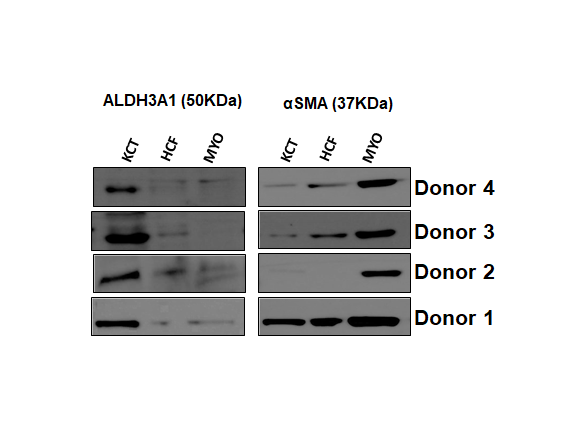

Supplement: Supplementary file 1 [file ijms-23-02572-s001.zip › Supplementary Figure S1.tif]

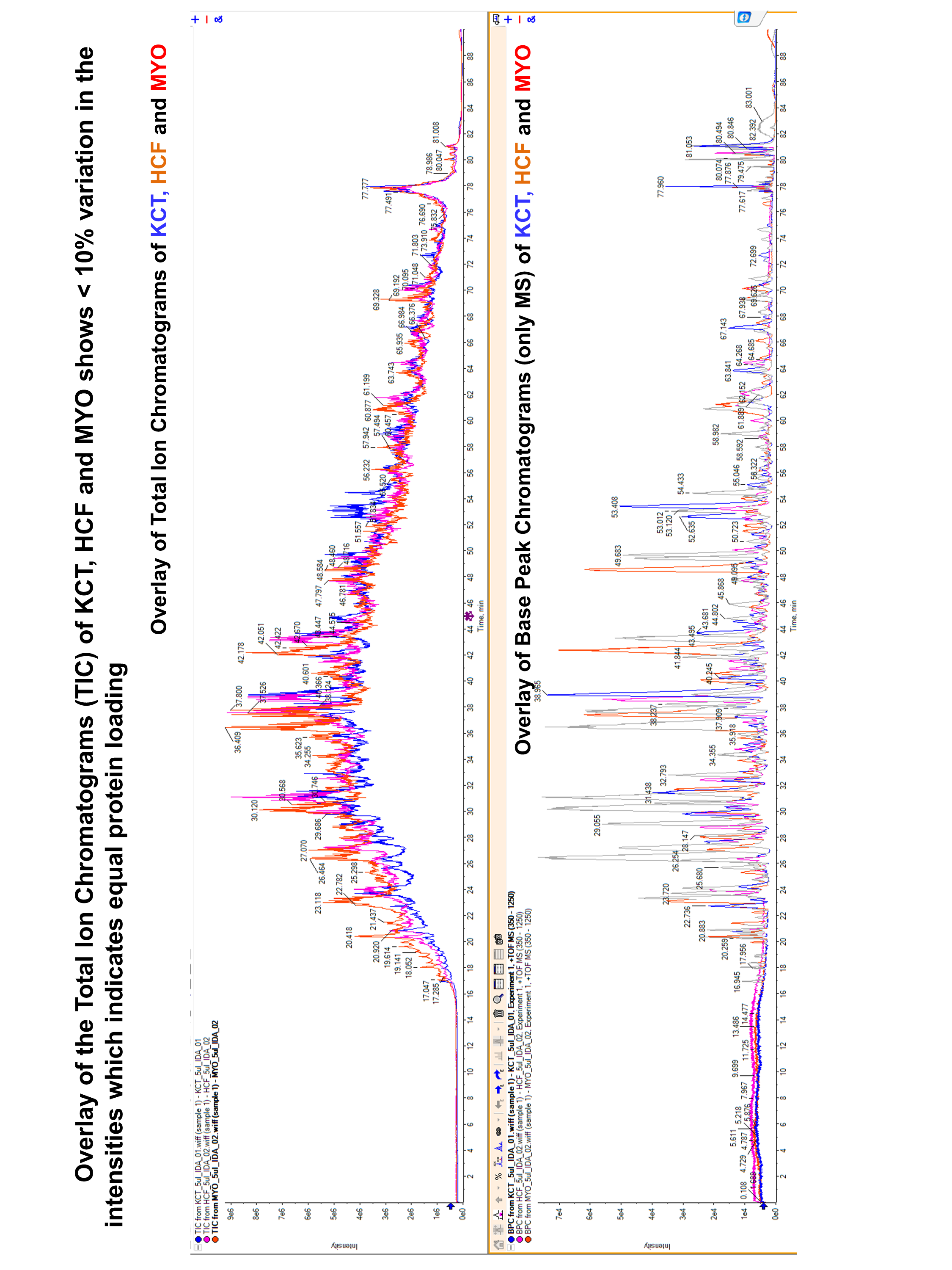

Supplement: Supplementary file 1 [file ijms-23-02572-s001.zip › Supplementary Figure S2.tif]

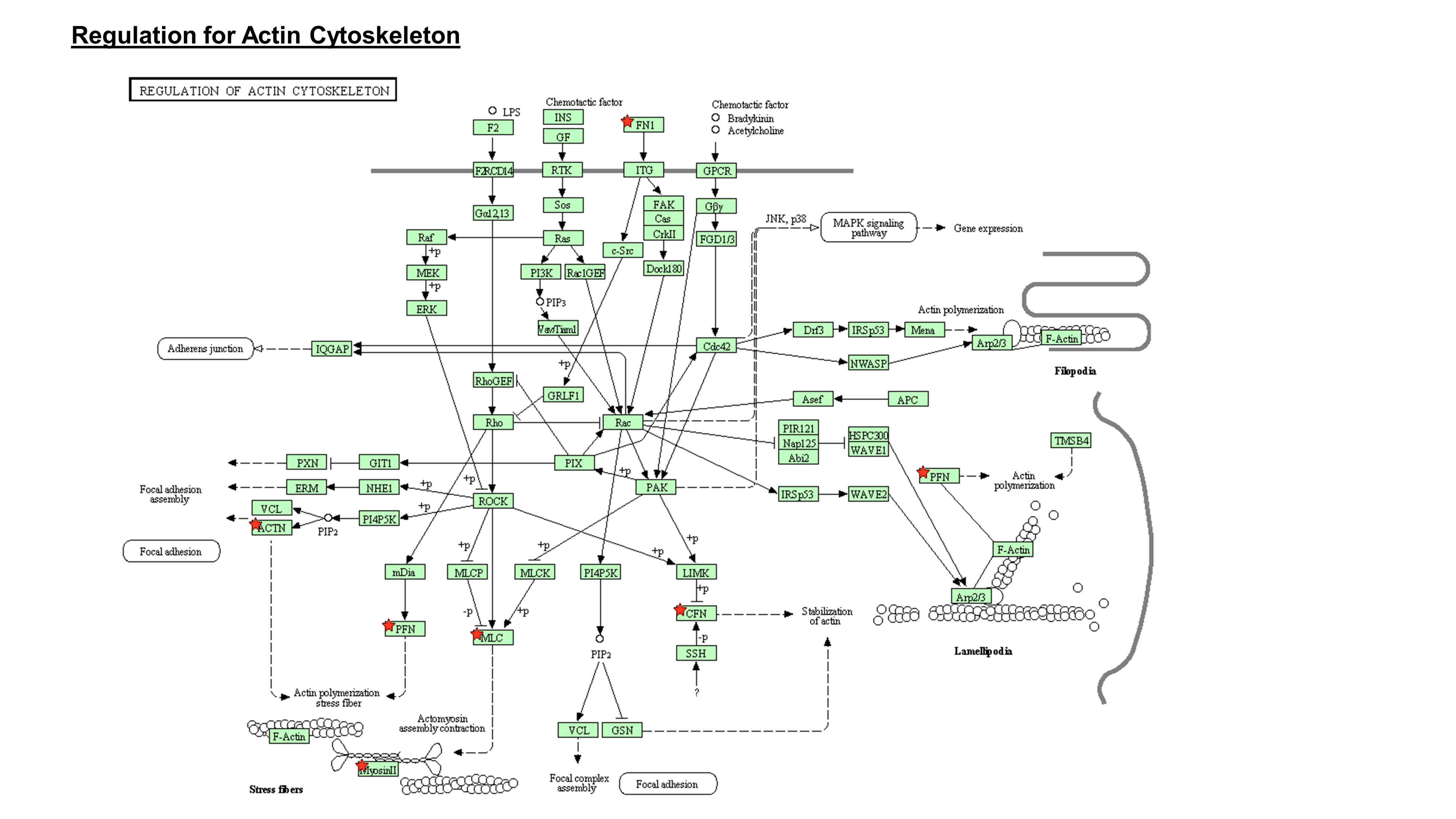

Supplement: Supplementary file 1 [file ijms-23-02572-s001.zip › Supplementary Figure S3.tif]

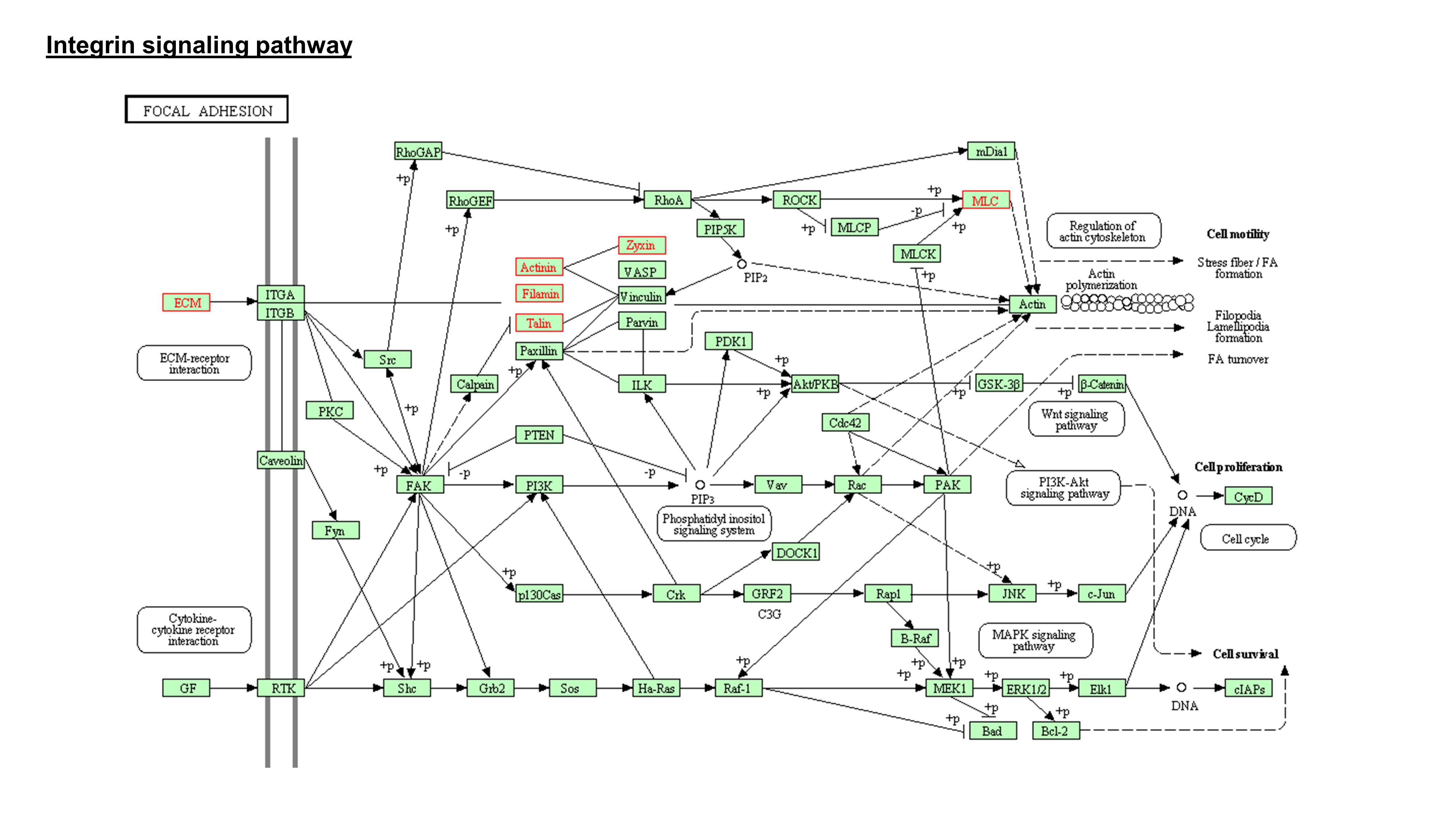

Supplement: Supplementary file 1 [file ijms-23-02572-s001.zip › Supplementary Figure S4.tif]
